# Effect of service experience, engagement and satisfaction on the future intentions of customers of a sports services

**DOI:** 10.1016/j.heliyon.2023.e17850

**Published:** 2023-07-03

**Authors:** García-Pascual Fernando, Parra-Camacho David, Aguado-Berenguer Sergio

**Affiliations:** Physical Education and Sport, University of Valencia, Valencia, Spain

**Keywords:** Management, Fitness centre, Engagement, Customer behaviour, Customer satisfaction

## Abstract

This research proposes a management model for a sports centre, through the variables of customer experience, satisfaction, engagement and its influence on the loyalty of its users. This work is carried out with the aim of providing sports managers with information that will enable them to improve the viability of their service. A sample of 378 customers of the sports service was used, 208 men and 170 women from a private sports centre. First, the psychometric properties of the scales used were obtained by means of a confirmatory factor analysis (CFA), followed by an analysis of their reliability and validity, and then the model fit was established using structural equations. Finally, the Sobel test was used to determine whether user satisfaction was mediated. Significant direct relationships between the constructs were found to exist, as well as an indirect effect of satisfaction within the model analysed. This model presented a goodness of fit, as well as the reliability analyses of the scales presented results supported by the literature. The results of this research demonstrate the mediating effect of satisfaction on the relationship between service experiences and customers' future commitment and intentions. It is important to know the experiences of customers through the sports service as it allows managers to develop precise strategies and actions to improve these experiences and raise the levels of commitment to the service, and consequently to strengthen the loyalty of customers for a long time.

## 1 Introduction

Sports organisations have been hit hard as a result of COVID-19, as it meant a very sharp slowdown in one of the sector's best moments. According to the European Health & Fitness Market 2022 study [1] the revenue received by the European fitness market in 2021 was 1.8 billion euros less than in 2020, although the number of customers increased. In Spain, between 2019 and 2021, the revenue of this market decreased by almost 25%, in the same trend was the number of users, losing these sports organisations almost 10% of participants. This demonstrates the importance of this sector in the economy, and how sports organisations must reorganise and establish management models that help to better understand the behaviours of customers and how, through their experiences with the service, they establish a greater commitment and loyalty to it.

These behaviours, such as loyalty, have been extensively analysed within the context of sports centre management [2]. Multiple variables have also been investigated in this field, such as perceived service quality [3], satisfaction [4], user behaviours [5], emotions [6]. All these variables analysed, have served to provide managers of these sports services with more tools that allow them to know the perceptions of users. Based on customers' experiences with the service and how they affect customers' future behaviour towards the service. Therein lies the importance of establishing management models that allow these sports managers to have detailed information on all the variables analysed and therefore allow them to establish strategies for positive behaviour towards the sports service in the future.

Therefore, the aim of this work is to develop a management model for sports centres, where the variables of customer experience with the service and the commitment (engagement) that users acquire once they have used the service are introduced, and what influence they have, together with other variables, on the future behaviour of customers, and to what degree loyalty towards the service is strengthened. On the other hand, to find out what role satisfaction can play as a mediating variable in the relationship between the variables and the customer's future intentions.

In this research, after the introduction, the theoretical framework based on the existing related literature and the suggested hypotheses are shown. Next, the method used is specified, developing the sample obtained, the procedure, the measuring instrument and finally the data analysis. Subsequently, the results obtained from the work are shown, as well as their relationship with the existing literature, in the discussion section. Finally, the conclusions obtained are presented, as well as the management proposals, limitations and future lines of research that could be derived from the research.

## 2 Theoretical framework

### 2.1 Optimal experience theory

In order to understand users' feelings of commitment to the service, the theory of optimal experience [7] is used. The subjective well-being of people is a state that has been analysed a lot in recent times [8,9], as it analyses constructs that lead to situations of positive affect. Positive psychology, which focuses on well-being, tries to indicate the value of a life, in the most satisfying way possible [10] by focusing on strength and optimal functioning [11]. Within this psychology, we find the construct of optimal experience theory or flow theory introduced by Csikszentmihalyi [7], defining it as the state expressed by people, of optimal experience, when they are involved in what they are doing and find it fun, even being an experience of such enjoyment that people will do it even at a higher cost for the simple fact of doing it. This perspective of experience is characterised by intrinsic motivation, positive reinforcement, clear goals, among others. This author characterises the creation of meaning as the main idea of this theory. These states of optimal experience or flow are related in the long term to positive results, such as increased engagement [12]. Engagement would be located in one of these constructs that facilitates positive feelings.

### 2.2 Management of sports services. Fitness centres

Any service offered is subject to constant analysis and evaluation by consumers, including sports consumers [13,14]. This is why this sector has always been analysed from the perceptions of the customers who hire and use it. However, the intangibility of these services provides an evaluation based on the experiences through said service in order to shape future behaviour towards it. This service experience has been widely studied in different contexts [15,16]. Meyer and Schwager [17] justify that service experience builds customers' cognitive, behavioural and emotional capacity in their response to the service. According to Zomerdijk and Voss [18], organisations are beginning to attach importance to service experiences, and thus as a consequence, boost customer loyalty.

This experience with the service can occur directly, initiated by the customer when establishing a relationship with the company in question, and indirectly, by having unplanned knowledge of the service, through conversations, news, etc. Knowing such customer experiences gives a broad and concrete way these relationships with the purchased service [19], but there are also other variables within the management models of these sports services, which facilitate a better understanding of the perceptions of them. Within sport services, models have always been established that analyse them based on intangible aspects such as satisfaction or perceived value [20,21], both of which are understood as evaluations subsequent to the acquisition of the service, thus helping in a complementary way to better understand the experiences of users, and how it would influence their future behaviours towards the service. Customer satisfaction is understood as the subjective evaluation made as a post-choice cognitive judgement [22]. Customer satisfaction of sport services has been a variable widely analysed in these contexts [[23], [24], [25]].

These variables that analyse the perceptions of customers of sports services offer values that allow us to know the state of the service, but it is also important to know what influence they have on the commitment to it, and thus be able to establish different actions that lead to raising the degree of commitment.

Engagement, or interaction, captures these perceptions of a sense of belonging and loyalty to the service, emotional aspects that determine the commitment of users who come to the sports centre. Van doorn et al. [26] define engagement as the behaviour of customers towards the brand or service, beyond the purchase. In the literature on sport management, specifically in sports centres, there is no literature that argues the relationship of this variable with the different management models that can be found. This variable has been extensively analysed in the context of sport social media [27,28] and sport events [29,30].

Being able to analyse all the perceptions of the members who uses these sports services, has one purpose, retention. Trying to achieve a loyal and faithful user to the service is one of the premises of the management of these sports centres, thus ensuring the viability of these companies. The future intentions of customers is one of the main variables analysed in these contexts [5,31]. Kincaid [32] describes consumer loyalty as consumer behaviour that, through experience, leads to the purchase of products, even if this is not the most rational decision. Hence the importance of creating positive experiences in these sports centres, and achieving a stable commitment on the part of the customer that leads to a positive and lasting loyalty.

### 2.3 Customer experience and service engagement

Knowing the experiences of customers who use a service is an analysis that allows us to obtain the subjective perceptions at a given time about the contracted service [33]. Due to the dynamism experienced by the fitness sector, in its continuous evolution, different variables are increasingly analysed in order to achieve the objective perception of customers. The service experience is a variable that is widely analysed within the context of sports organisations [34,35], as it allows sports managers to know in a more detailed way their closest perceptions of the service. This customer experience is oriented from the utilitarian view of the service, or utilitarian consumption [36], i.e. what is actually desired from that service. They conclude that the service experience establishes customers' emotional and cognitive behaviours towards the service. García-Fernández and Sañudo Corrales [37], in their work analysing the experiences of an age group in private sports centres, conclude that emotional experiences lead to greater loyalty behaviour in these members. Within this work, the variable that measures the degree of commitment of the customer to the sports service and how this influences loyalty is introduced. Engagement is a variable that has been little analysed in sports centres, in terms of the commitment acquired by the customer towards the sports service.

H1 Service experience has a significant relationship with customer engagement.

### 2.4 Customer experience, engagement and satisfaction

Knowing the impressions of members, on the part of the company, after acquiring a service, is fundamental for preparing future strategies and continuing to strengthen the relationship with the user. There are studies in which the utilitarian version of consumption offered by the service experience produces emotions of security and tranquillity that give the customer satisfaction [15,38,39]. Therefore, they produce emotionally stable environments, leading to pleasure and consequently customer satisfaction [40]. Within the management of sport organisations, the analysis of perceived service quality has been prioritised as cognitive aspects of members [2,41]. Likewise, experience becomes the predecessor of customer satisfaction, thus leading organisations to articulate the signals or perceptions given by customers in the purchasing process, and thus be able to provide them with a satisfactory experience [42]. Customer satisfaction, which measures the subjective experience with the service, has become one of the most analysed variables within sport organisations [21,23,43]. Kim and Ling [44], in their work in fitness centres, state that customer experiences have an impact on satisfaction.

Therefore, knowing the relationship between the emotional and behavioural perceptions of clients, through their experiences after purchasing a service, and the degree of satisfaction achieved by customers is a relationship that should be known and measured.

Depending on the level of customer satisfaction, it will also depend on their future behaviour towards the service. Likewise, engagement is a variable that has also been adapted within the management models of these sports services [45,46]. Teik [47], in his work analysing fitness centres in Malaysia, concludes that engagement is a means to achieve member satisfaction. This author also argues that the engagement that occurs in the customer towards the sports service helps to establish values such as socialisation, building communities.

H2 Experiences with the service acquired influence customer satisfaction.

H3 The engagement of customers towards the sports service has a direct relationship with customer satisfaction.

### 2.5 Customer satisfaction and future customer behaviour

Knowing the future behaviour of members is the main objective of sports services, in order to know the loyalty of customers towards the service, and to be able to develop strategies that allow the viability of the sports centre. Finding satisfied users in fitness centres leads to creating loyal customers, since satisfaction is a clear antecedent of customer's future intentions [48]. Within the field of sports centre management, there are many studies that have analysed the relationship between both variables [5,[49], [50], [51], [52]]. Gonçalves et al. [53], in a study carried out in fitness centres, concludes that keeping members satisfied is key for any sports service, as this satisfaction can lead to the user repeating the experience.

H4 Overall customer satisfaction significantly influences customer's future behaviours.

### 2.6 Customer experience, engagement, satisfaction and future customer intentions

Interpreting the perceptions of the members of a sports service has been the subject of analysis for some time [31,33]. Knowing the evaluations that customers give when interacting with the service is fundamental for the future of the service. These evaluations, analysed through customer experiences, have a significant influence on the relationship between the service and the customer in the future. In turn, member experiences lead to a certain degree of satisfaction, which is also related to customer's future behaviour towards the service. Some studies have shown that satisfaction has an indirect effect on the relationship between customer's perceptions and experiences with the service and their future intentions towards the service [54,55]. These works analyse how customer satisfaction makes facilitating perceptions more significant in member's future behaviours with the service. Howat et al. [56], demonstrated in their work in public aquatic centres how satisfaction had a mediating role between customer's perceptions and their behavioural intentions. This helps to understand how member's perceptions and experiences of sport services guide customers behavioural and decision-making processes.

Engagement, understood as the user's commitment to the service, is a variable that has been analysed a lot in recent times in the context of sport management [57,58]. So et al. [59], argued that engagement is significant because of its effects on customer's future intentions. Members with high levels of engagement intend to have positive interactions with the service [60] and consequently with behavioural loyalty [61]. According to Kumar et al. [62], in order to improve the viability of organisations, it is necessary to understand the aspects related to behavioural loyalty, including engagement. Within the context of fitness centres, customer satisfaction also plays an important role and it is in these processes where customer behaviours are analysed. Memon et al. [63], in their work finds that there is an indirect effect of customer satisfaction on the relationship between customer engagement and loyalty. Within the context of sport services, there are also papers that have argued for the mediation of satisfaction in the relationship between engagement and future customer behaviours [64,65].

H5 There is a mediation of satisfaction between customer experience and future intentions.

H6 There is a mediation of satisfaction between engagement and future intentions.

## 3 Method

### 3.1 Participants

A total of 411 questionnaires were collected, of which 33 were eliminated as they were incomplete, resulting in a final sample of 378 valid questionnaires. A total of 208 men (55%) and 170 women (45%) were counted, with an average age of 34.5 years. Of this sample, sixty (16%) had primary education, one hundred and sixty-nine members (45%) had secondary education, and one hundred and fourty-nine of the users who participated in the research (39%) had a university education. Also, around 70% were physically active 2 or more times a week.

### 3.2 Procedure

The questionnaires were collected between March and June 2019, in a private sports centre in the province of Valencia, Spain. During that time, and at the entrance of this sports facility, in person, different collection times were scheduled in order to obtain the perceptions of a representative part of the users of the service. The average time taken by customers to complete the questionnaire was between 8 and 9 min. Purposive convenience sampling was used. Customers voluntarily agreed to participate and were assured that the data obtained would be anonymous and confidential. This study was carried out at the University of Valencia. This university, in its Department of Ethics and Human Research Committee, does not consider consent necessary to conduct an opinion survey on a professional situation, or topic with different aspects.

### 3.3 Instrument

The measurement instrument that was used to collect the perceptions of the members of the sports service, a first block was formed by 4 constructs with a total of 33 indicators and the second block included demographic variables such as age, gender, level of studies and sports frequency. The 33 indicators in the first block had a 5-point Likert-type scale as a response option (1 means strongly disagree and 5 means strongly agree).

The scale measuring the service experience perceived by the customers of the sports centre is composed of 15 indicators adapted from the Klaus & Maklan [66] scale corresponding to the constructs of reliability (6 indicators), professionalism (5 indicators) and response capacity (4 indicators). This instrument was previously validated, thus confirming correct psychometric properties [38].

Customer satisfaction was measured by two indicators taken from Ref. [67]. Previous studies also confirmed the adequate psychometric properties of the scale [23].

Customer engagement towards the service was measured through the Sport Engagement Scale [68]. This scale was adapted to measure engagement in the sport context in Spain and consists of 12 indicators grouped into 3 constructs (vigour, dedication and absorption). Previous studies within the sport context confirmed the psychometric properties of the scale [69].

Finally, the scale analysing the customers future intentions towards the service, taken from Zeithaml et al. [70], consisted of 4 indicators. The psychometric properties of this scale were confirmed in recent studies [50].

### 3.4 Statistical analysis

To test the psychometric properties of the scales, a confirmatory factor analysis (CFA) was performed. This analysis was carried out using EQS 6.4 program, applying the Robust Maximum Likelihood Estimation (MLE) method in order to correct for the possible absence of multivariate normality, using statistics such as Satorra Bentler's χ2 [71]. Thus, for the assessment of the overall fit, use was made of different goodness-of-fit indices recommended in the literature [72], such as the Chi-square significance and its robust correction provided by Satorra-Bentler (*S*–B χ^2^) [73]. Other coefficients were also calculated to test the adequacy of the proposed models, such as the χ^2^ ratio and its degrees of freedom (χ2/df [74]), with values of less than five being acceptable [75]. On the other hand, the coefficients of the robust goodness-of-fit indices of the proposed model corresponding to the Non-Normed Fit Index (NNFI), the Comparative Fit Index (CFI) and the Incremental Fit Index (IFI) were tested. For these indicators, values above 0.90 are considered a good fit [76]. Finally, the root mean square error of approximation (RMSEA) is shown, with scores below 0.08 [77] being necessary to consider a good fit.

On the other hand, the reliability of the scales was analysed using Cronbach's Alpha, Composite Reliability (CF) and the Extracted Variance Measure (AVE) [78]. To test for convergent validity, the significance of the factor loadings of the indicators in their respective dimension and the associated *t*-test values were analysed [79]. Discriminant validity, which is concerned with the clear distinction between any pair of constructs, was assessed using the method suggested by Fornell & Larcker [80]. This method admits discriminant validity if the square root of the AVE value of a given factor is greater than the correlation coefficients between the factor and any other factor of the proposed scale. The value of correlations between pairs of constructs was also analysed, with values below 0.85 being recommended [72].

After checking the validity and reliability of the scales, the fit of the proposed structural equation model was tested using different fit indices mentioned (*S*–B χ^2^; χ2/df; RMSEA; NNFI; CFI; IFI). The structural model was evaluated using the R2 estimates, standardised beta coefficients (β) and significance level (t-value). Finally, the Sobel test was used to test the mediating effect of overall satisfaction between perceived impacts and future intentions.

## 4 Results

### 4.1 Evaluation of the measurement model

First, the psychometric properties of the indicators of the customer experience perception scales, engagement, satisfaction and future intentions were analysed. Table 1 shows the values of the mean, standard deviation, item-total correlation corrected item-total, alpha if the item is removed, skewness and kurtosis. The values of the corrected item-total correlation coefficient were higher than the cut-off point recommended by the literature (≥0.30). Also, the values of skewness and kurtosis are acceptable for most of the variables as they are lower than 3.0 [81].

**Table 1:** Mean, standard deviation, asymmetry and kurtosis of the indicators of the scales.

Item	M	SD	rjx	α-x	A	K
**Customer experience**						
*Factor 1 - Reliability*						
FE1 - I have confidence in the competence of CDM staff.	4.19	.94	.69	.85	−1.37	2.12
FE2 - The whole process with CDM has been easy	4.27	.89	.59	.86	−1.21	1.24
FE3 - CDM is interested in me as a long-term customer	3.64	1.22	.66	.85	−.61	−.43
FE4 - My past experience with CDM makes me trust and remain with this centre.	3.96	1.08	.78	.83	−.89	.18
FE5 – In my dealings with CDM it has always been easy to get what I wanted	3.86	1.16	.70	.84	−.80	−.25
FE6 - CDM provides me with individualised advice and information.	4.19	.94	.64	.86	−.70	−.38
*Factor 2 – Response capacity*						
RC1 - CDM offers flexibility and adapts to my needs.	3.76	1.20	.68	.84	−.60	−.56
RC2 - CDM keeps me informed	3.69	1.19	.68	.84	−.46	−.82
RC 3 - CDM is a trustworthy company that provides security	3.60	1.25	.71	.83	−.91	.20
RC 4 - The staff at CDM are friendly	3.93	1.11	.63	.85	−1.25	.93
RC 5- CDM has offered me proper treatment and attention in dealing with my complaints.	4.15	1.08	.73	.83	−.96	−.07
Factor 3- *Professionalism*						
P1 – I feel comfortable being a customer of the CDM	4.20	.94	.77	.85	−1.11	.75
P2 - CDM adapts quickly to my needs	3.75	1.16	.79	.83	−.61	−.50
P3 – I choose CDM over other similar sports centres	3.88	1.18	.73	.86	−.88	−.12
P4 - CDM staff understand my needs	3.86	1.12	.72	.86	−.81	−.03
**Engagement**						
*Factor 1 - Absortion*						
EG1 - I ′m very persistent when practising sport in the CDM.	4.05	.99	.69	.79	−.74	−.30
EG2 - I feel strong and engaged in my physical activity.	4.17	.91	.71	.78	−.84	−.05
EG3 - When I wake up in the morning, I look forward to training at the CDM	3.85	1.15	.66	.80	−.87	.04
EG4 - I can train for a long time in the CDM.	4.00	1.10	.64	.81	−.95	.16
*Factor 2 - Dedication*						
D1 - While doing physical activity in the CDM I feel inspired.	3.98	1.03	.79	.83	−.71	−.38
D2 - When I come to the CDM, I am enthusiastic about my physical activity.	4.04	.98	.79	.82	−.79	−.03
D3 - I am proud of the sport I do at CDM	4.15	.95	.79	.83	−1.00	.60
*Factor 3 - Vigour*						
V1 – I am engrossed in my physical activity when I come to the CDM.	3.96	1.03	.68	.85	−.85	.28
V2 - Time passes very quickly when I am training at CDM.	4.06	.99	.70	.85	−.89	.25
V3 - When I go to the CDM I am happy because I am focused on my physical activity.	4.10	.92	.74	.84	−.79	.02
V4 – I ′m oblivious to everything going on around me when I train at the CDM.	3.82	1.13	.64	.87	−.77	−.10
V5 - At CDM, I am immersed in my sporting practice.	4.09	.90	.77	.83	−.75	−.04
**Satisfaction**						
S1 – I ′m happy with the experiences I have had at CDM.	4.08	.97	.84	–	−.91	.32
S2 – I ′m satisfied with my experiences doing sport at CDM.	4.19	.92	.84	–	−1.05	.55
**Future Intentions**						
FI1 – Probably, I will continue to attend the facility next year/year.	4.13	1.04	.79	.93	−1.12	.61
FI2 – I will recommend coming to this facility to anyone who asks me.	4.00	1.12	.89	.90	−.99	.25
FI3 – I will encourage friends and relatives to sign up for this facility.	3.98	1.12	.90	.90	−1.01	.33
FI4 – I normally speak highly of the services offered by this facility.	4.06	1.07	.81	.93	−1.04	.34

Note: M = Mean; SD= Standard deviation; rjx = item-total correlation corrected item-total; α-x = alpha if the items is removed; A = Assimetry; K=Curtosis.

After testing the item properties, a CFA was performed for the scales under study. The CFA showed adequate goodness-of-fit indices for the model: significant chi-square (χ2 = 1306.59, gl = 467, p < .01), a norm chi-square value (χ2/gl = 2.80) of less than 5 and the RMSEA index showed a value of 0.047 (Confidence interval = 0.042-0.051), lower than 0.08. In the same line, the rest of the indices show a good fit of the model, as they presented values higher than 0.90: NNFI = 0.94; CFI = 0.95; and IFI = 0.95.

To analyse reliability, Cronbach's alpha, composite reliability (CR) and Average Variance Extracted (AVE) measures were observed (see Table 2). Cronbach's alpha values were above 0.70 for all constructs and scales, as recommended by the literature. This criterion was also met for the CR values, with values ranging from 0.84 to 0.94. Finally, for the AVE values, all constructs were found to have values above the 0.50 recommended by the literature (see Table 3).

**Table 2:** Factor loadings, composite reliability and average variance extracted values de las dimensions.

Item	λ	α	CR	AVE
**Customer Experience**				
*Factor 1 - Reliability*		**.87**	.88	.54
FE1	.71			
FE2	.66			
FE3	.72			
FE4	.83			
FE5	.78			
FE6	.71			
*Factor 2 – Response capacity*		.86	.86	.56
RC 1	.76			
RC 2	.70			
RC 3	.79			
RC 4	.69			
RC 5	.79			
*Factor 3- Professionalism*		.88	.89	.66
PR1	.85			
PR2	.84			
PR3	.78			
PR4	.78			
**Engagement**				
*Factor 1 - Absortion*		.84	.84	.57
EG1	.75			
EG2	.76			
EG3	.78			
EG4	.74			
*Factor 2 - Dedication*		.89	.89	.73
D1	.85			
D2	.87			
D3	.83			
*Factor 3 - Vigour*		.87	.88	.59
V1	.74			
V2	.80			
V3	.82			
V4	.67			
V5	.81			
**Satisfaction**		.91	.91	.84
S1	.92			
S2	.91			
**Future Intentions**		.94	.94	.79
FI1	.83			
FI2	.93			
FI3	.94			
FI4	.85			

Note. λ = Factor loadings; α = Cronbach's alpha; CR = composite reliability; AVE = average variance extracted.

**Table 3:** Correlations between dimensions.

	F1	F2	F3	F1	F2	F3	S	FI
*Factor 1 - Reliability*	*0.74*							
*Factor 2 – Response capacity*	.84**	*0.75*						
Factor 3- *Professionalism*	.83**	.83**	*0.82*					
Factor 1 - *Absortion*	.49**	.48**	.52**	*0.76*				
Factor 2- *Dedication*	.55**	.55**	.62**	.73**	*0.85*			
Factor 3 - *Vigour*	.51**	.49**	.57**	.68**	.77**	*0.77*		
Satisfaction	.71**	.69**	.75**	.55**	.64**	.64**	*0.91*	
Future intentions	.76**	.71**	.77**	.55**	.61**	.58**	.80**	*0.89*

Note: **p < .01. The diagonal offers the values of the √AVE.

To analyse convergent validity, the *t*-test values associated with the factor loadings of the items were found to be greater than 1.96 (p < .05), ranging from 12.18 to 22.12 for the indicators of the scale of perceived positive impacts, and between 13.69 and 24.26 for the items of the scale of negative impacts. The factor loadings of all items were also found to be above 0.50. In terms of discriminant validity, on the one hand, we found that the correlation between the two constructs was below 0.85 (see Table 4). On the other hand, we found that the square root of the AVE was higher than the correlation between pairs of constructs, also fulfilling this criterion, except between the constructs of the engagement scale.

**Table 4:** Results of the proposed model and mediation tests.

Hypothesized Paths	Direct effects		Mediation test	
	Standardised Coefficient	t-valor	Standardised Coefficient	t-valor
H1: EXP - ENG	.68 **	8.10		
H2: EXP - SAT	.64 **	9.57		
H3: ENG - SAT	.33 **	5.54		
H4: SAT - FI	.88 **	17.10		
*Mediation check*				
H5 y H6: Step 1. Independent variables - Dependent variable				
EXP – FI			.75**	13.50
ENG - FI			.28**	−6.12
H5 y H6: Step 2. Independent variables - Mediating variable				
EXP - SAT			.69**	15.09
ENG - SAT			.43**	−6.78
H5 y H6: Step 3–4. Independent variables and mediating variable - Dependent variable				
EXP – FI			.61**	10.91
ENG - FI			.15**	3.60
SAT - FI			.49**	10.78

Note: EXP= Service Experience; ENG = Engagement; SAT= Satisfaction; FI= Future Intentions; **p < .01.

### 4.2 Relationship between constructs and mediating effect

To test the mediating effect of overall satisfaction, the four steps outlined by Baron and Kenny [82] and the application of Sobel's test were followed. Table 4 shows the standardised coefficients and the *t*-test value for each of the three causal relationship models conducted to test the mediating effect. These models show the results of the relationships between the constructs under study: service experience, engagement, satisfaction and future intentions.

**Fig. 1:**
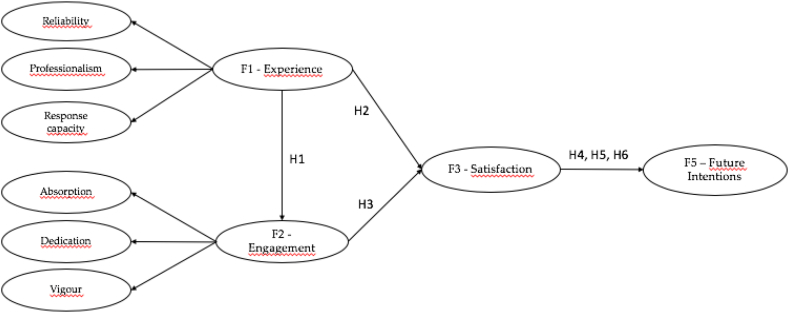
Theorical model.

The proposed model (Fig. 1) presents a good fit of the main reference indicators: χ^2^/df = 2.85; RMSEA = 0.048 (CI = 0.043-0.052); NNFI = 0.94; CFI = 0.94; IFI = 0.94. The results allow us to confirm H1 as a positive significant relationship was observed between customer experience and engagement (β = .68, t = 8.10, p < .01). H2 and H3 were also confirmed as the statistically significant influence of customer experience (β = 0.64, t = 9.57, p < .01) and engagement (β = 0.33, t = 5.54, p < .01), respectively, on customer satisfaction was found. H4 was also confirmed as there is a significant relationship between satisfaction and customer future intentions (β = .88, t = 17.10, p < .01).

To test step 1 and 2 proposed by Baron and Kenny [82], it is necessary to test for a statistically significant relationship between the independent variables (customer experience and engagement) and the mediating variable (satisfaction) and dependent variable (future intentions). Secondly, it should be checked that when the mediating variable is introduced the relationship between the independent variables and the dependent variable should be significantly smaller (steps 3 and 4). Full mediation is established when the standardised coefficients of the independent variables in step 1 are significant and the same coefficients are not significant in step 4 [82]. Otherwise, assuming all four conditions hold, partial mediation is supported [82,83].

The Step 1 and Step 2 models explained 64% (R2 = 0.64) and 66% (R2 = 0.66), respectively. In Step 3 and 4, the results confirm the significant relationship between satisfaction (mediator) and future intentions (β = .49, t = 10.78, p < .01), proving that the other two independent variables in this model are significantly related, but reduce their coefficient by introducing the mediator variable as an additional independent variable.

Finally, to test for the existence of mediation effects, the Sobel test was applied. This test allowed us to test for the existence of a mediation effect of customer satisfaction between service experience and future service intentions (z = 8.87, p < .01) and between engagement and future intentions (z = 7.45, p < .01). This effect was partially mediated because the coefficients of the independent variables in step 4 were significantly related to future intentions, so full mediation cannot be established [82,83]. Ultimately, the results confirm H5 and H6 as the mediating effect of customer satisfaction on the relationship between service experience and engagement and future intentions was verified.

## 5 Discussion and conclusions

The models that define the management of sports centres all pursue the same objective: to have a clear profile of the perceptions of the members who come to the service. Knowing these perceptions will help to carry out and programme actions that allow for greater user loyalty and extend the viability of the sports service.

This work analyses the management model of private sports centres, in the relationship between the constructs that measure the service experience and engagement and future customer behaviour, as well as the mediating role of customer satisfaction, One of the main results of this work is the important role of customer satisfaction in this model as a mediator of the relationship between user experiences and engagement with future customer behaviour. Satisfaction has been a much analysed variable both in its direct and indirect effects with other constructs that confirm fitness centre management models. Although within the literature, its direct relationship with other variables, such as perceived value [25,50], perceived quality of service [84] or emotions [85,86], has always been more analysed. However, within the context of these sport services, few studies [54,63] delve into the role of satisfaction as a mediator of future consumer behaviours.

Furthermore, this work shows that satisfaction with the sports service significantly predicts customer future intentions, a relationship that has been widely analysed in the context [24,87] of fitness centre management [24,87]. According to Zairi [88], a satisfied customer will share his or her experiences with six people, but a dissatisfied consumer will share these negative experiences with up to ten people. Therefore, these sports organisations are concerned about having satisfied members in order to obtain customers with high levels of loyalty to the service, and thus reduce the abandonment rates of the same.

This research shows how the constructs that measure service experience obtain positive correlations with users' future intentions. Eskiler and Safak [89] in their research on the quality of customer experience in fitness centres, concluded that those customers who have positive experiences tend to continue using the service, even recommending it to others.

It also shows how engagement has a significant effect on future sports service behaviors. Bozkurt et al. [90], argued that customer engagement has a positive impact on user behavioural engagement, such as recommendations, influence, among others.

Therefore, within the field of sports centre management, paying attention to aspects such as the professionalism of the service or the reliability perceived by the member, as well as the degree of commitment, will increase satisfaction and, to a greater extent, customer loyalty to the sports service.

This study provides the literature with significant and mediating relationships between the constructs that make up the management models in the field of sports centres. Therefore, analysing the mechanisms that promote these model relationships, such as service experience or commitment to future customer behaviour, is essential in order to understand the degree of influence that analysing these customer perceptions and expectations has on the future of these sports services.

### 5.1 Theoretical and practical implications

The results of this research conclude that satisfaction is a variable with a mediating role between the relationship of consumer experience and engagement with customer's future behaviours towards the sports service, thus providing the managers of these sports centres with tools to strengthen and reinforce member loyalty.

Through the management models of these fitness centres, including the one analysed in this research, the managers of these services are given a justification of the importance of analysing and understanding the perceptions and experiences of users, as these will guide the decision-making process of these customers over time.

Therefore, this paper provides the managers of these sports services with an informative orientation that allows them to interpret the perceptions and behaviours of their members, in order to be able to apply strategies that allow them to establish stronger links in a short period of time.

This work has again shown the strong relationship between customer satisfaction based on the confirmation of their experiences and positive behaviour towards the sports service. Therefore, these experiences and a strong commitment to the service will suggest positive future intentions through customer satisfaction.

Currently within the fitness centre environment there is a very high level of competition with a wide range of sporting offerings, therefore this model tries to clarify beyond the daily transactions, which constructs can influence the flow of members to be denser, reinforcing the feeling of loyalty, through attractive experiences. Therefore, sports managers of these sports centres should not expect high and positive levels of future customer behaviour if there is no significant satisfaction with the service. Therein lies the importance of knowing what actions or strategies should be applied in this context to reinforce these expectations and positively modify customer satisfaction. In this sense, obtaining satisfied members does not only reinforce the customers feeling of loyalty, but will also allow them to positively recommend the sports service to their environment.

### 5.2 Limitations and future lines of research

This work has been carried out using a non-probabilistic sample, so it cannot be generalised to all members of private sports centres. Therefore, in future research, it would be advisable to expand the number of sports centres, as this study has only focused on a single centre. It would also be advisable to increase the sample and broaden the context, using members of public sports centres, jointly analysing customer's perceptions.

On the other hand, it would be advisable to explore the impact of variables such as price on the constructs analysed in this study.

In future research to reinforce and better understand customer's perceptions and expectations, and thus provide more in-depth knowledge to sports managers, other constructs could be included in this model, such as brand [91,92], previous purchase behaviour [31] or also psychological variables [62,93] where the emotional component of the member during the interaction with the sports service is analysed.

## Author contribution statement

Fernando García-Pascual: Conceived and designed the experiments; Wrote the paper.

David Parra-Camacho: Performed the experiments; Analysed and interpreted the data.

Sergio Aguado: Contributed reagents, materials, analysis tools or data.

## Data availability statement

No data was used for the research described in the article.

## Additional information

No additional information is available for this paper.

## Declaration of competing interest

The authors declare that they have no known competing financial interests or personal relationships that could have appeared to influence the work reported in this paper.

## References

[1] El fitness continua creciendo en Espana, Deloitte Spain. (n.d.). https://www2.deloitte.com/es/es/pages/technology-media-and-telecommunications/articles/el-negocio-del-fitness.html (accessed March 28, 2020).

[2] Avourdiadou S., Theodorakis N.D. (2014). The development of loyalty among novice and experienced customers of sport and fitness centres. Sport Manag. Rev..

[3] Pradeep S., Vadakepat V., Rajasenan D. (2020). The effect of service quality on customer satisfaction in fitness firms. Manag. Sci. Letters.

[4] Chiu W., Won D., Bae J. (2019). Customer value co-creation behaviour in fitness centres: how does it influence customers' value, satisfaction, and repatronage intention?. Managing Sport and Leisure.

[5] Foroughi B., Iranmanesh M., Gholipour H.F., Hyun S.S. (2019). Examining relationships among process quality, outcome quality, delight, satisfaction and behavioural intentions in fitness centres in Malaysia. IJSMS.

[6] García-Pascual F., Prado-Gascó V., Alguacil M., Valantine I., Calabuig-Moreno F. (2020). Future intentions of fitness center customers: effect of emotions, perceived well-being and management variables. Front. Psychol..

[7] Mihaly Csikszentmihalyi (1991). Flow, the psychology of optimal experience. Am. J. Psychother..

[8] Brim O.G., Ryff C.D., Kessler R.C. (2019).

[9] Ecclestone K., Hayes D. (2009). Changing the subject: the educational implications of developing emotional well‐being. Oxf. Rev. Educ..

[10] Loureiro S., Panchapakesan P. (2017).

[11] García-Renedo M., Llorens S., Cifre E., Salanova M. (2006). https://redined.educacion.gob.es/xmlui/handle/11162/69067.

[12] Shernoff D.J., Csikszentmihalyi M., Schneider B., Shernoff E.S., Csikszentmihalyi M. (2014). Applications of Flow in Human Development and Education: the Collected Works of Mihaly Csikszentmihalyi.

[13] ángel Mañas Rodríguez M., Giménez Guerrero G., Muyor Rodríguez J.M., Martínez Tur V., Moliner Cantos C.P. (2008). Los tangibles como predictores de la satisfacción del usuario en servicios deportivos. Phys. Scr., T.

[14] Nuviala Nuviala R., Teva-Villén M.R., Pérez-Ordás R., Grao-Cruces A., Tamayo Fajardo J.A., Nuviala Nuviala A. (2015). Segmentación de usuarios de servicios deportivos (Segmentation of sport services users). Retos.

[15] Bolton R.N., Lemon K.N., Bramlett M.D. (2006). The effect of service experiences over time on a supplier's retention of business customers. Manag. Sci..

[16] Veiga E., Beauchamp T.B., Bomfim E.L., Takashi H. (2019). Measuring customer experience in service: a systematic review. Serv. Ind. J..

[17] Meyer C., Schwager A. (2007). Understanding customer experience. Harv. Bus. Rev..

[18] Zomerdijk L.G., Voss C.A. (2010). Service design for experience-centric services. J. Serv. Res..

[19] Chen C.-C.V., Chen C.-J. (2017). The role of customer participation for enhancing repurchase intention. Manag. Decis..

[20] Clavel San Emeterio I., Iglesias-Soler E., Gallardo L., Rodriguez-Cañamero S., García-Unanue J. (2016). A prediction model of retention in a Spanish fitness centre. Manag. Sport and Leisure.

[21] Ferreira Barbosa H., García-Fernández J., Pedragosa V., Cepeda-Carrion G. (2021). The use of fitness centre apps and its relation to customer satisfaction: a UTAUT2 perspective. Int. J. Sports Mark. Spons..

[22] Brown G., Smith A., Assaker G. (2016). Revisiting the host city: an empirical examination of sport involvement, place attachment, event satisfaction and spectator intentions at the London Olympics. Tourism Manag..

[23] García-Pascual F., Molina N., Mundina J. (2019). Influencia de la satisfacción y el valor percibido sobre el “Word of Mouth” en los usuarios de centros deportivos. SPORT TK-Revista Euro Am. de Ciencias del Deporte.

[24] Howat G., Assaker G. (2013). The hierarchical effects of perceived quality on perceived value, satisfaction, and loyalty: empirical results from public, outdoor aquatic centres in Australia. Sport Manag. Rev..

[25] Nuviala A., Grao-Cruces A., Pérez-Turpin J.A., Nuviala R. (2012). Perceived service quality, perceived value and satisfaction in groups of users of sports organizations in Spain. Kinesiology.

[26] van Doorn J., Lemon K.N., Mittal V., Nass S., Pick D., Pirner P., Verhoef P.C. (2010). Customer engagement behavior: theoretical foundations and research directions. J. Serv. Res..

[27] Aichner T. (2019). Football clubs' social media use and user engagement. MIPS.

[28] Naraine M. (2019). Follower segments within and across the social media networks of major professional sport organizations. Sport Market. Q..

[29] Huettermann M., Uhrich S., Koenigstorfer J. (2019). Components and outcomes of fan engagement in team sports: the perspective of managers and fans. J. Global Sport Manag..

[30] Jones C., Byon K., Huang H. (2019). Service quality, perceived value, and fan engagement: case of shanghai formula one racing. Sport Market. Q..

[31] Yi S., Lee Y.W., Connerton T., Park C.-Y. (2021). Should I stay or should I go? Visit frequency as fitness centre retention strategy. Manag. Sport and Leisure.

[32] Kincaid J.W. (2003).

[33] Calabuig F., Núñez-Pomar J., Prado-Gascó V., Añó V. (2014). Effect of price increases on future intentions of sport consumers. J. Bus. Res..

[34] Baena-Arroyo M.J., Gálvez-Ruiz P., Sánchez-Oliver A.J., Bernal-García A. (2016). The relationship among service experience, perceived value and behavioural intentions of customers in a group fitness class. Rev. Psicol. Deporte.

[35] Dias C., Ferreira A., Pereira A.R. (2019). Examining the relationship between perceived service quality, satisfaction, and renewal intention in Portuguese tness centers. Rev. Psicol. Deporte.

[36] Chitturi R., Raghunathan R., Mahajan V. (2007). Form versus function: how the intensities of specific emotions evoked in functional versus hedonic trade-offs mediate product preferences. J. Market. Res..

[37] García-Fernández J., Sañudo Corrales B. (2013). Las experiencias de servicio en clientes mayores de 50 años y su influencia en la lealtad en centros de fitness privados. Kronos.

[38] Chang W.-L., Huang L.-Y. (2016). Measuring service experience: a utility-based heuristic model. Serv Bus.

[39] Chernev A. (2004). Goal–attribute compatibility in consumer choice. J. Consum. Psychol..

[40] Ladhari R. (2007). The effect of consumption emotions on satisfaction and word-of-mouth communications. Psychol. Market..

[41] Lagrosen S., Lagrosen Y. (2007). Exploring service quality in the health and fitness industry. Manag. Serv. Qual..

[42] Berry L.L., Carbone L.P., Haeckel S.H. (2002).

[43] Calabuig-Moreno F., Quintanilla-Pardo I., Mundina-Gómez J. (2008). La calidad percibida de los servicios deportivos: diferencias según instalación, género, edad y tipo de usuario en servicios náuticos. (The perception of service quality in sport services: differences according to sport facility, gender, age and user type in nautical services). rev. int. cienc. deporte..

[44] Kim C.B., Ling T.C. (2017). The influence of the service quality and outcome quality on the member overall satisfaction. Global Bus. Manag. Res.: Int. J..

[45] Feng W., Tu R., Hsieh P. (2020). Can gamification increases consumers' engagement in fitness apps? The moderating role of commensurability of the game elements. J. Retailing Consum. Serv..

[46] Torkzadeh S., Zolfagharian M., Yazdanparast A., Gremler D.D. (2022). From customer readiness to customer retention: the mediating role of customer psychological and behavioral engagement. Eur. J. Market..

[47] Teik D.O.L. (2015). Enhancing the experience of needs satisfaction through service engagement: a case of commercial fitness centers in Malaysia. J. Global Scholars of Market. Sci..

[48] Tsiros M., Mittal V. (2000). Regret: a model of its antecedents and consequences in consumer decision making. J. Consum. Res..

[49] Calabuig F., Crespo J., Mundina J. (2012). Effect of perceived cost, service quality and satisfaction on future intentions of spectators. Estud. Econ. Apl..

[50] García-Fernández J., Gálvez-Ruíz P., Fernández-Gavira J., Vélez-Colón L., Pitts B., Bernal-García A. (2018). The effects of service convenience and perceived quality on perceived value, satisfaction and loyalty in low-cost fitness centers. Sport Manag. Rev..

[51] Haro-González M., Pérez-Ordás R., Grao-Cruces A., Nuviala R., Nuviala A. (2018). Female users of unisex fitness centres and of fitness centres exclusive for women: satisfaction. Int. J. Sports Mark. Spons..

[52] Silla A., Calabuig F., Añó V. (2014). Emociones, satisfacción e intenciones futuras de los usuarios de actividades dirigidas de un centro deportivo. J. Sports Econ. Manag..

[53] Gonçalves C., Biscaia R., Correia A., Diniz A. (2014). An examination of intentions of recommending fitness centers by user members. Motriz: Rev. Educ. Fis..

[54] Clemes M.D., Brush G.J., Collins M.J. (2011). Analysing the professional sport experience: a hierarchical approach. Sport Manag. Rev..

[55] Yoshida M., James J.D. (2010). Customer satisfaction with game and service experiences: antecedents and consequences. J. Sport Manag..

[56] Howat G., Crilley G., Mcgrath R. (2008). A focused service quality, benefits, overall satisfaction and loyalty model for public aquatic centres. Manag. Leisure.

[57] Behnam M., Pyun D.Y., Doyle J.P., Delshab V. (2020).

[58] García-Fernández J., Gálvez-Ruiz P., Sánchez-Oliver A.J., Fernández-Gavira J., Pitts B.G., Grimaldi-Puyana M. (2020). An analysis of new social fitness activities: loyalty in female and male CrossFit users. Sport Soc..

[59] So K.K.F., King C., Sparks B.A., Wang Y. (2016). Enhancing customer relationships with retail service brands: the role of customer engagement. J. Serv. Manag..

[60] Hapsari R., Clemes M.D., Dean D. (2017). The impact of service quality, customer engagement and selected marketing constructs on airline passenger loyalty. Int. J. Quali. Serv. Sci..

[61] Bowden J.L.-H. (2009). The process of customer engagement: a conceptual framework. J. Market. Theor. Pract..

[62] Kumar V., Sharma A., Shah R., Rajan B. (2013). Establishing profitable customer loyalty for multinational companies in the emerging economies: a conceptual framework. J. Int. Market..

[63] Memon M.A., Salleh R., Mirza M.Z., Cheah J.-H., Ting H., Ahmad M.S. (2019). Performance appraisal satisfaction and turnover intention: the mediating role of work engagement. Manag. Decis..

[64] Alexandris K., Zahariadis P., Tsorbatzoudis C., Grouios G. (2004). An empirical investigation of the relationships among service quality, customer satisfaction and psychological commitment in a health club context. Eur. Sport Manag. Q..

[65] Javadein S.R.S., Khanlari A., Estiri M. (2008). Customer loyalty in the sport services industry: the role of service quality, customer satisfaction, commitment and trust. Journal of Human Sciences.

[66] Klaus P., Maklan S. (2012). EXQ: a multiple‐item scale for assessing service experience. J. Serv. Manag..

[67] Hightower R., Brady M.K., Baker T.L. (2002). Investigating the role of the physical environment in hedonic service consumption: an exploratory study of sporting events. J. Bus. Res..

[68] Guillén F., Martínez-Alvarado J.R. (2014).

[69] Waleriańczyk W., Hill A.P., Stolarski M. (2022). A re-examination of the 2x2 model of perfectionism, burnout, and engagement in sports. Psychol. Sport Exerc..

[70] Zeithaml V.A., Berry L.L., Parasuraman A. (1996). The behavioral consequences of service quality. J. Market..

[71] Chou C.-P., Bentler P.M., Satorra A. (1991). Scaled test statistics and robust standard errors for non-normal data in covariance structure analysis: a Monte Carlo study. Br. J. Math. Stat. Psychol..

[72] Kline R.B. (2015). https://books.google.es/books?id=Q61ECgAAQBAJ.

[73] Satorra A., Bentler P.M. (1994). Latent Variables Analysis: Applications for Developmental Research.

[74] Wheaton B., Muthén B., Alwin D.F., Summers G.F. (1977). Assessing reliability and stability in panel models. Socio. Methodol..

[75] Byrne B.M. (2013). https://books.google.es/books?id=8vHqQH5VxBIC.

[76] MacCallum R.C., Austin J.T. (2000). Applications of structural equation modeling in psychological research. Annu. Rev. Psychol..

[77] Browne M.W., Cudeck R., Bollen K.A., Long J.S. (1993). Testing Structural Equation Models.

[78] Hair J.F., Black W.C., Babin B.J., Anderson R.E. (2014). https://www.amazon.es/Multivariate-Data-Analysis-Joe-Hair/dp/0138132631.

[79] Anderson J., Gerbing D. (1988). Structural equation modeling in practice: a review and recommended two-step approach. Psychol. Bull..

[80] Fornell C., Larcker D.F. (1981). Evaluating structural equation models with unobservable variables and measurement error. J. Market. Res..

[81] Chou C.-P., Bentler P.M. (1995). Structural Equation Modeling: Concepts, Issues, and Applications.

[82] Baron R.M., Kenny D.A. (1986). The moderator-mediator variable distinction in social psychological research: conceptual, strategic, and statistical considerations. J. Pers. Soc. Psychol..

[83] Prayag G., Hosany S., Nunkoo R., Alders T. (2013). London residents' support for the 2012 Olympic Games: the mediating effect of overall attitude. Tourism Manag..

[84] Polyakova O., Ramchandani G. (2020). Perceived service quality among regular users of gyms in public sports centres in the UK. Managing Sport and Leisure.

[85] Molina N., Mundina J., García-Pascual F., Alejos E. (2016). El efecto de la experiencia de servicio emocional en las intenciones futuras del usuario de centros deportivos. Rev. Psicol. Deporte.

[86] Pedragosa V., Biscaia R., Correia A. (2015). The role of emotions on consumers' satisfaction within the fitness context. Motriz: Rev. Educ. Fis..

[87] Fernández-Martínez A., Dueñas-Dorado L.A., Teva-Villén M.R., Nuviala A. (2021). Consolidation, stages of change, and loyalty among users of public sports and health services aged 12–16. Int. J. Environ. Res. Publ. Health.

[88] Zairi M. (2000). Managing customer satisfaction: a best practice perspective. TQM Mag..

[89] Eskiler E., Safak F. (2022). Effect of customer experience quality on loyalty in fitness services, physical culture and sport. Stud. Res..

[90] Bozkurt S., Gligor D., Gligor N. (2022). Investigating the impact of psychological customer engagement on customer engagement behaviors: the moderating role of customer commitment. J Market Anal.

[91] Alguacil M., García-Fernández J., Calabuig F., Gálvez-Ruiz P. (2022). How can the management of fitness centres be improved through corporate image and brand image?. Econ. Res.-Ekonomska Istraživanja.

[92] Mohsen Y., Hussein H.M., Mahrous A.A. (2018). Perceived service value, customer engagement and brand loyalty in health care centres in Egypt. MMI (Med. Microbiol. Immunol.).

[93] Molina García N., Crespo-Hervás J., García Pascual F. (2018).

